# Reproducibility of Neurite Orientation Dispersion and Density Imaging (NODDI) in rats at 9.4 Tesla

**DOI:** 10.1371/journal.pone.0215974

**Published:** 2019-04-29

**Authors:** Patrick McCunn, Kyle M. Gilbert, Peter Zeman, Alex X. Li, Michael J. Strong, Ali R. Khan, Robert Bartha

**Affiliations:** 1 Center for Functional and Metabolic Mapping (CFMM), Robarts Research Institute, University of Western Ontario, London, Ontario, Canada; 2 Department of Medical Biophysics, University of Western Ontario, London, Ontario, Canada; 3 Molecular Medicine Research Group, Robarts Research Institute, University of Western Ontario, London, Ontario, Canada; 4 Department of Clinical Neurological Science, Robarts Research Institute, University of Western Ontario, London, Ontario, Canada; 5 Departments of Psychiatry and Medical Imaging, University of Western Ontario, London, Ontario, Canada; Dalhousie University, CANADA

## Abstract

**Purpose:**

Neurite Orientation Dispersion and Density Imaging (NODDI) is a diffusion MRI (dMRI) technique used to characterize tissue microstructure by compartmental modelling of neural water fractions. Intra-neurite, extra-neurite, and cerebral spinal fluid volume fractions are measured. The purpose of this study was to determine the reproducibility of NODDI in the rat brain at 9.4 Tesla.

**Methods:**

Eight data sets were successfully acquired on adult male Sprague Dawley rats. Each rat was scanned twice on a 9.4T Agilent MRI with a 7 ± 1 day separation between scans. A multi-shell diffusion protocol was implemented consisting of 108 total directions varied over two shells (b-values of 1000 s/mm^2^ and 2000 s/mm^2^). Three techniques were used to analyze the NODDI scalar maps: mean region of interest (ROI) analysis, whole brain voxel-wise analysis, and targeted ROI analyses (voxel-wise within a given ROI). The coefficient of variation (CV) was used to assess the reproducibility of NODDI and provide insight into necessary sample sizes and minimum detectable effect size.

**Results:**

CV maps for orientation dispersion index (ODI) and neurite density index (NDI) showed high reproducibility both between and within subjects. Furthermore, it was found that small biological changes (<5%) may be detected with feasible sample sizes (n < 6–10). In contrast, isotropic volume fraction (IsoVF) was found to have low reproducibility, requiring very large sample sizes (n > 50) for biological changes to be detected.

**Conclusions:**

The ODI and NDI measured by NODDI in the rat brain at 9.4T are highly reproducible and may be sensitive to subtle changes in tissue microstructure.

## Introduction

Diffusion weighted magnetic resonance imaging (dMRI) is a powerful magnetic resonance modality that provides a wealth of information regarding tissue microstructure, from which structural connectivity and pathological changes within the brain can be inferred [1,2]. As different microstructures predictably retard diffusion, the apparent diffusion of molecules combined with the angle of an applied diffusion gradient provides an indirect measure of neuroanatomy [3]. The most commonly used dMRI technique is diffusion tensor imaging (DTI). For DTI, a series of pulsed-gradient, spin–echoes are used to produce a 3x3 symmetric matrix modelling Gaussian diffusion (3). Most commonly, DTI characterizes the overall water diffusion within a given voxel by measuring mean diffusivity (MD) and the degree of directionality of the principle component of this diffusion, through fractional anisotropy (FA). This technique has been utilized for many years and has provided valuable insights into the effects of disease, as well as neurological and physiological processes [4–8].

However, subtle tissue diffusion characteristics may be missed in DTI because the method lacks the specificity to identify unique microstructural environments. For example, DTI cannot distinguish between distinct processes such as the loss of structural integrity and neural remodelling, and as a result provides an inherently vague and limited model of neuroanatomy [9,10]. Several more sophisticated dMRI models have been developed to overcome the limitations of DTI such as Q-Ball imaging [11], CHARMED [12], diffusion kurtosis imaging [13], oscillating gradient diffusion MRI [14], and more recently neurite orientation dispersion and density imaging (NODDI) [15]. NODDI examines neurite morphology by specifically probing the unique diffusion patterns within three separate microstructural environments: intra-neurite, extra-neurite, and CSF compartments [15].

Diffusion patterns within the brain may be separated into three distinct microstructural environments: highly restricted within neurites (intra neurite compartment), hindered diffusion near neurites (extra-neurite compartment) and free diffusion within the CSF compartment [16]. A carefully designed diffusion weighting scheme in an MRI pulse sequence combined with NODDI modelling is used to produce scalar maps indicating the volume fraction contribution of each compartment to the full diffusion signal. The intra-neurite space is modelled as cylinders of zero radius (modelling highly restricted diffusion perpendicular to neurites and free diffusion parallel to neural tracts) dispersed according to the Watson distribution (ranging from heavily dispersed to entirely parallel) while the extra-neurite space is modelled as Gaussian anisotropic diffusion [17]. Lastly the CSF compartment is modelled with Gaussian isotropic diffusion [15].

The NODDI acquisition incorporates a multi-shell protocol that leads to a multi-compartmental diffusion MR signal. Previous work has shown that the use of 2 shells (each shell corresponding to a subset of diffusion weightings known as b-values) combined with several b = 0 images, is sufficient to obtain NODDI scalar maps in-vivo [15]. These images are reconstructed based on the Stejskal-Tanner equations for a pulsed gradient spin-echo (PGSE) experiment, and the total signal determined to be the sum of the individual contributions from three non-exchanging tissue compartments (intra-cellular, extra-cellular, and CSF compartments) [15]. From this signal, quantitative scalar image maps may be reconstructed yielding the following NODDI metrics: Orientation Dispersion Index (ODI), Neurite Density Index (NDI), and Isotropic volume fraction (IsoVF). Additionally, by ensuring one shell conforms to DTI acquisition standards (e.g. a single shell with > six directions of b = 1000 s/mm^2^ and one b = 0 volume) [1], it is possible within a single scan to obtain standard diffusion tensor metrics such as fractional anisotropy (FA) and mean diffusivity (MD). It is commonly recommended that DTI and NODDI be acquired simultaneously and analyzed together [10]. While MD and FA are routine measures obtained in diffusion imaging, the addition of the ODI, NDI, and IsoVF scalar maps can provide a more specific analysis of complex neuroanatomy [9,10]. ODI characterizes the angular variation and spatial configuration of neurite structures. NDI represents the fraction of tissue that comprises axons or dendrites (also referred to as intra-neurite volume fraction). Extra-neurite fraction may be reconstructed as 1- NDI, and as such provides does not provide unique information above NDI. IsoVF represents the CSF water fraction [15].

Previous use of NODDI has focused largely on the feasibility, reproducibility, and application to human imaging at field strengths up to 3 Tesla [18–24]. Specifically, it was shown that NODDI metrics were significantly dependent on field strength [25]. Many pre-clinical studies use rodent models to study neuro-pathological processes requiring extremely small voxel sizes relative to that used in human MRI. Image signal to noise ratio (SNR) is directly proportional to voxel size and to main magnetic field strength. Therefore, the use of ultra-high field strengths combined with strong imaging gradients helps to achieve adequate SNR for diffusion modelling at the image resolution required in rodent models [26]. While the feasibility of NODDI at 9.4 Tesla has been shown [27], we are aware of no studies that have explored reproducibility in rodent models at 9.4 Tesla. As ultra-high field MRI, and specifically pre-clinical rodent MRI, faces many unique challenges such as increased magnetic field inhomogeneities and physiological noise [28], it is important to carefully define reproducibility in the context of ultra-high field rodent imaging. Thus, our specific objective was to determine the reproducibility of the three most commonly derived NODDI metrics (ODI, NDI and IsoVF) at 9.4 Tesla in the rat brain. This information is crucial for the planning of future studies involving rat models of neurodegenerative disease or neurological injury.

## Methods

### Subjects

Ten adult male Sprague Dawley rats were scanned twice on separate days with 7 ± 1 days between scans. Sample sizes were chosen to reflect common practice in pre-clinical imaging studies. On the day of the scans, anesthesia was initiated by placing the animals in an induction chamber with 4–5% isoflurane and an oxygen flow rate of 1–1.5 L/min. Following induction, isoflurane was maintained between 1.5–2.5% with an oxygen flow rate of 1–1.5 L/min through a custom-built nose cone. All animal procedures were approved by the University of Western Ontario Animal Use Subcommittee and were consistent with guidelines established by the Canadian Council on Animal Care.

### Imaging

All images were acquired using a 31 cm bore 9.4 T Agilent small animal MRI scanner at the Centre for Functional and Metabolic Mapping at the University of Western Ontario. Images were acquired with an eight-channel receive coil used in conjunction with a 2-channel transmit coil. The receive coil consisted of eight loops adhered to the inner surface of a conformal helmet that was adjustable in width to accommodate varying head sizes. Low input-impedance preamplifiers were used to reduce inter-element coupling. The transmit coil was comprised of two overlapped rectangular loops mounted on an inverted U-shaped former. The coil design and optimization followed that built for marmoset imaging [29], but with dimensions optimized for rat imaging.

The NODDI diffusion encoding scheme was incorporated into a centric-ordered spin echo echo-planar-imaging (EPI) acquisition pulse sequence (number of shots = 4, number of averages = 2, 25 slices with slice thickness = 500 μm, FOV 40 x 40 mm, matrix size 160 x 160, resulting in-plane resolution = 250 × 250 μm, TE = 25 ms, TR = 5.0 s). Two averages were used, rather than increased diffusion directions, to ensure adequate SNR in the higher b-value shell for NODDI reconstruction. As it is recommended that NODDI be used in conjunction with standard DTI metrics (FA and MD) [10] we chose a b-value of 1000 s/mm^2^ for the inner shell. Following the work of Zhang *et*. *al*. [15], a second b-value of 2000 s/mm^2^ was chosen. Use of these b-values has been shown to produce reproducible values of NODDI specific metrics in human imaging at lower field strengths, and can be used to obtain standard DTI measures [15]. To sample q-space, we chose a scheme totaling 108 directions spread across two b-values, optimized according to Caruyer *et*. *al*. [30]. This sampling scheme allows for twice the number of directions in the higher b-value shell. Specifically, the outer shell consisted of 72 b-value = 2000 s/mm^2^ directions (gradient strength (G) = 339.1 mT/m, time between the start of the first and second diffusion pulse (Δ) = 14.44 ms, the duration of a single gradient pulse (δ) = 4.32 ms, TE = 25 ms and TR = 5.0s). The inner shell consisted of 36 b-value = 1000 s/mm^2^ directions (G = 169.6 mT/m, Δ = 14.44 ms, δ = 4.32 ms, TE = 25 ms and TR = 5.0s). Fifteen b = 0 s/mm^2^ were interspersed evenly throughout the acquisition and two preparation volumes were acquired at the beginning of each average but not used, resulting in a total imaging time of 83 minutes. A single reverse phase encoded b = 0 volume was acquired at the end of the diffusion sequence for subsequent use in TOPUP and EDDY (number of shots = 4, number of averages = 2, 25 slices with slice thickness = 500 μm, FOV 40 x 40 mm, matrix size 160 x 160, resulting in-plane resolution = 250 × 250 μm, TE = 25 ms, TR = 5.0 s). Anatomical images were also acquired for each subject within each session using a 3D fast low angle shot [31] pulse sequence (250-μm isotropic resolution, FOV 40 x 40 x 20 mm, matrix size = 160 x 160 x 50, TE = 5.0 ms, TR = 30.0 ms, total acquisition time = 7 min).

### Image processing

Images were pre-processed using fMRI Software Library (FSL, v.5.0.10, Oxford, UK). TOPUP [32] followed by EDDY [33] was used to correct for eddy current induced distortions as well as susceptibility-induced distortions. Brain masks were produced using the 3D Pulse Coupled Neural Network (PCNN) tool for Matlab [34]. The NODDI Matlab toolbox (available from the UCL Microstructure Imaging Group) was then used to produce maps of ODI, NDI, and IsoVF in diffusion space.

For each subject, the first volume in each diffusion data set (b = 0) was aligned with its corresponding anatomical images using a linear registration in FSL (FLIRT) [35]. The transformation matrix from the preceding step was then used to bring all NODDI scalar maps into anatomical space. Anatomical images were then aligned to the Waxholm Space Atlas Sprague Dawley template [36] using a linear transformation (FLIRT) followed by a non-linear transformation (FNIRT) [37] in FSL. FNIRT registration parameters were optimized for the registration of rodent images. While a quantitative analysis (such as Dice coefficient) of registration quality was not performed, anatomical images were visually inspected to ensure good registration quality. The Waxholm Space Atlas Sprague Dawley template includes binary masks for the relevant brain regions of interest in this study. Inverse transformation matrices from the preceding steps were used to bring these masks from template space into the anatomical image space of each rat. Each mask was eroded by 5% around the edges to avoid partial volume effects within a given ROI.

### Statistical analysis

Statistical analyses to examine measurement reproducibility were performed for the mean region of interest (ROI) analysis, the whole brain voxel-wise analysis, and the voxel-wise analysis within a given ROI. These three techniques were chosen as they represent the most common analysis techniques in neuroimaging studies. The ROI analysis focused on six different tissue regions: thalamus, corpus callosum, dentate gyrus, hippocampus, whole brain white matter, and whole brain grey matter. In both the ROI and voxel-wise analyses the scan-rescan reproducibility were characterized using the coefficient of variation (CV). CV was chosen as it reflects both the reproducibility and variability of these metrics as well as provides insight into necessary sample sizes and minimum detectable effect size. CVs were calculated between subjects and within subjects to quantify the between subject reproducibility and within subject reproducibility respectively. The between subject CV was calculated separately for the scan and rescan conditions as the group standard deviation divided by the mean values from subjects 1–8. These two CV values were then averaged for the mean between subjects CV in each case. The within subject CV was calculated as the standard deviation of the two scans divided by the mean value. The 8 within subjects CVs were then averaged to determine the mean within subject CV. Furthermore, the between subject CV was used to determine the minimum number of subjects needed per group to detect a defined biological effect. Similarly, the within subject CV was used to calculate the minimum detectable biological effect with a given number of subjects per group. The details of these calculations follow those presented in van Belle [38]. The minimum number of subjects and minimum detectable biological effect were both determined at a 95% significance level (*α* = 0.05) and power of 80% (1−*β* = 0.80).

## Results

**Fig 1:**
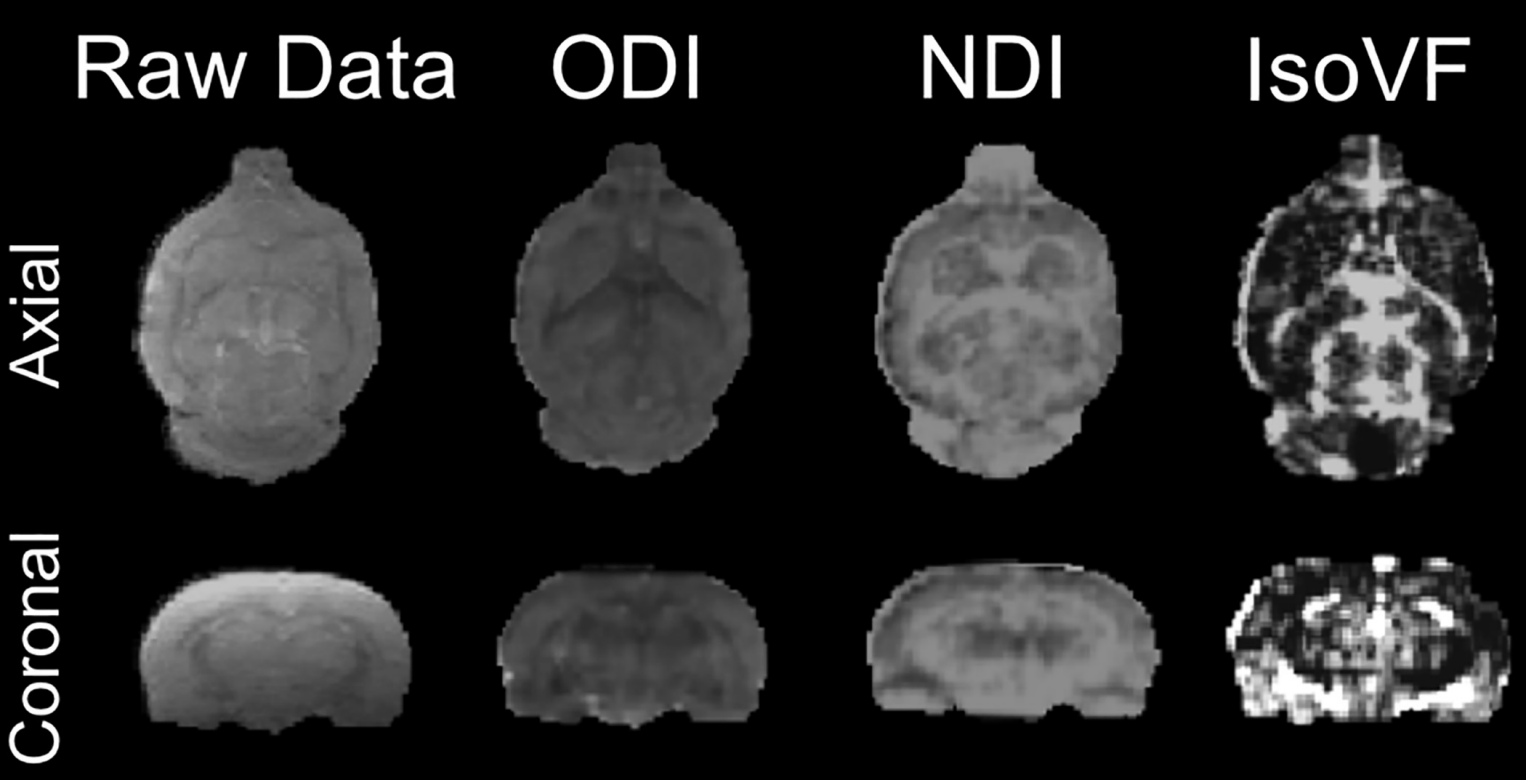
Representative in-plane cross sections from a single subject showing unprocessed raw diffusion image data (4 shot, centric ordered, 2 averages, 25 coronal slices with slice thickness = 500 μm, 250 × 250 μm in plane resolution, FOV 40 x 40 mm, matrix size = 160 x 160, TE = 25 ms, TR = 5.0 s), and corresponding scalar image maps of the following NODDI values: Orientation Dispersion Index (ODI), Neurite Density Index (NDI), and Isotropic Volume Fraction (IsoVF).

The minimum accepted average whole-brain SNR was 25 at b = 0 for each of the included data sets. Two data sets were removed from the analysis due to low SNR causing significant reconstruction bias. Therefore, data were successfully acquired and analyzed from eight subjects (age 102 ± 13 days at time of initial scan, weight 323 ± 37 g) at two separate time points. For each subject the re-scan time point was between six and eight days after the original scan. The time of day was not standardized for the scans. Fig 1 shows representative cross sections of raw diffusion data (b = 0) from a single subject, as well as scalar maps of ODI, NDI, and IsoVF.

### ROI analysis

**Fig 2:**
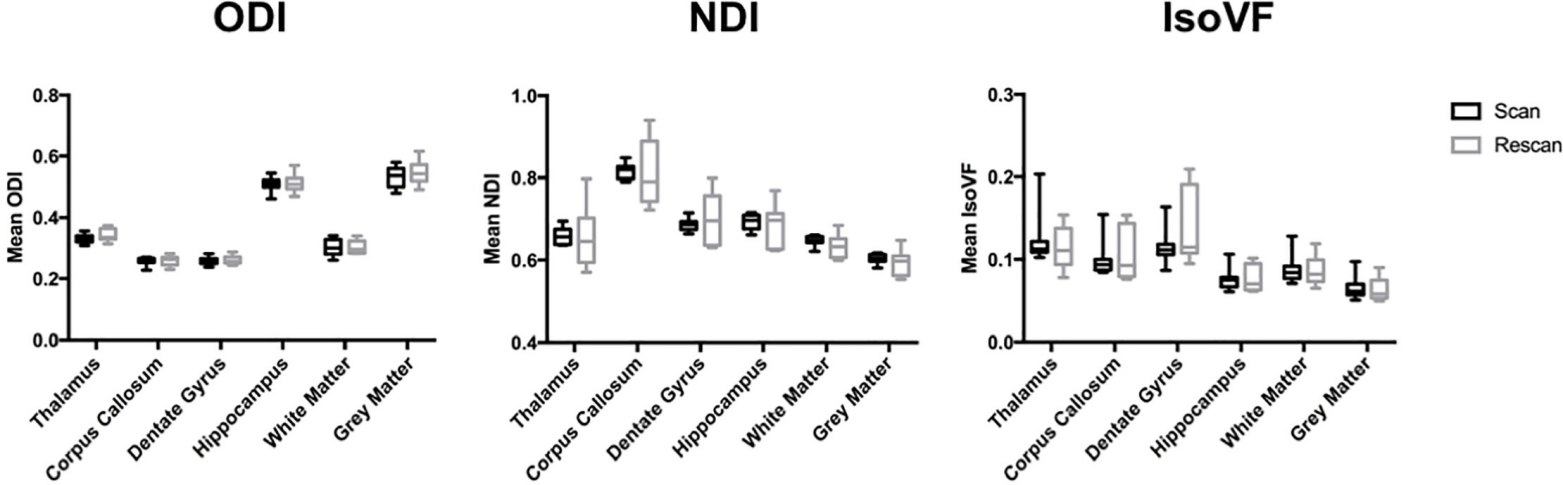
Region of interest (ROI) values for ODI, NDI and IsoVF in both the scan and rescan conditions for several representative brain regions. Each box represents the range from 25^th^ to 75^th^ percentile (Interquartile Range) with the median depicted by the line within the box. The whiskers shown extend to the maximum and minimum value of each measurement.

**Fig 3:**
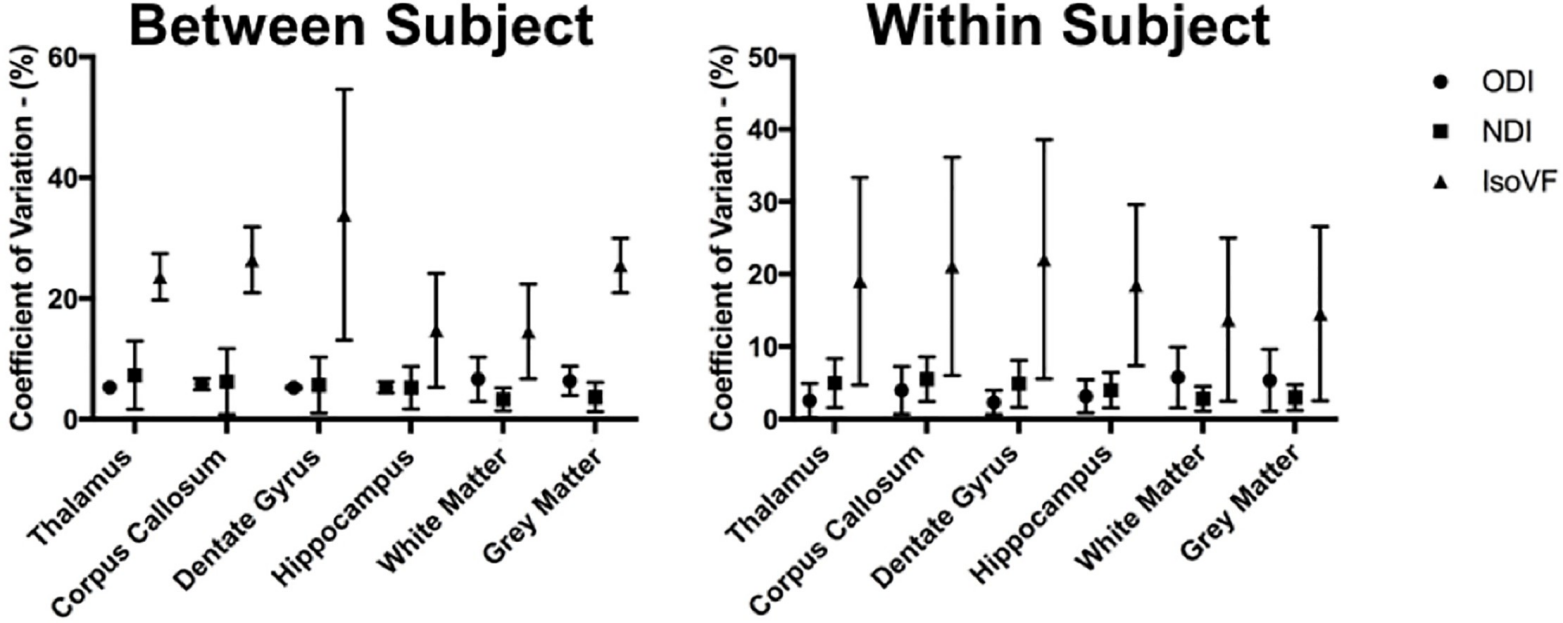
Mean coefficient of variation (CV) for each ROI. Values for the between subject condition represent the mean ± standard deviation within each ROI averaged over a scan-rescan protocol. Values for the within subject condition represent the mean ± standard deviation within each ROI averaged over the eight subjects.

Similar to previous human and rodent studies, higher average ODI values were observed within grey matter regions compared to that of white matter regions (Fig 2) as expected because neurites are more widely dispersed throughout grey matter [25,27]. NDI and IsoVF values were similar between white and grey matter. Mean between and within subject CV for ODI ranged from 4.0–9.2% within all ROIs, NDI ranged from 1.9–11.3%, while IsoVF ranged from 9.0–48.6% (Fig 3). In general, for each metric within a given ROI the mean between subject CV was higher than the within subject CV.

### Whole brain voxel-wise analysis

**Fig 4:**
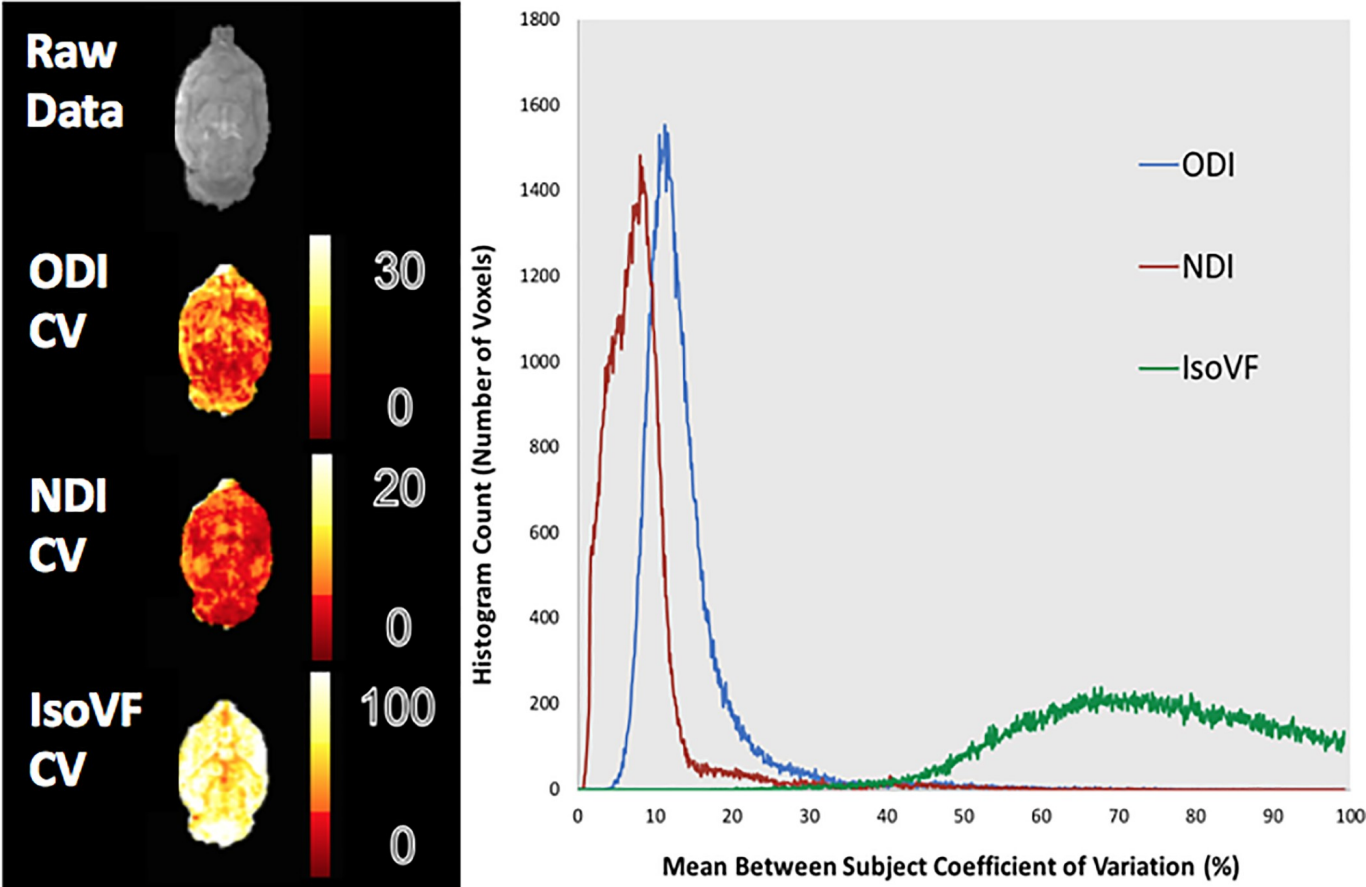
Whole brain average between subject CV maps and histogram. Values for the between subject condition represent the mean CV within each voxel for the scan and rescan conditions averaged over the two scans. The resulting histogram has been extracted from the averaged scans. Heat maps from a representative slice show the regional variation for each metric.

**Fig 5:**
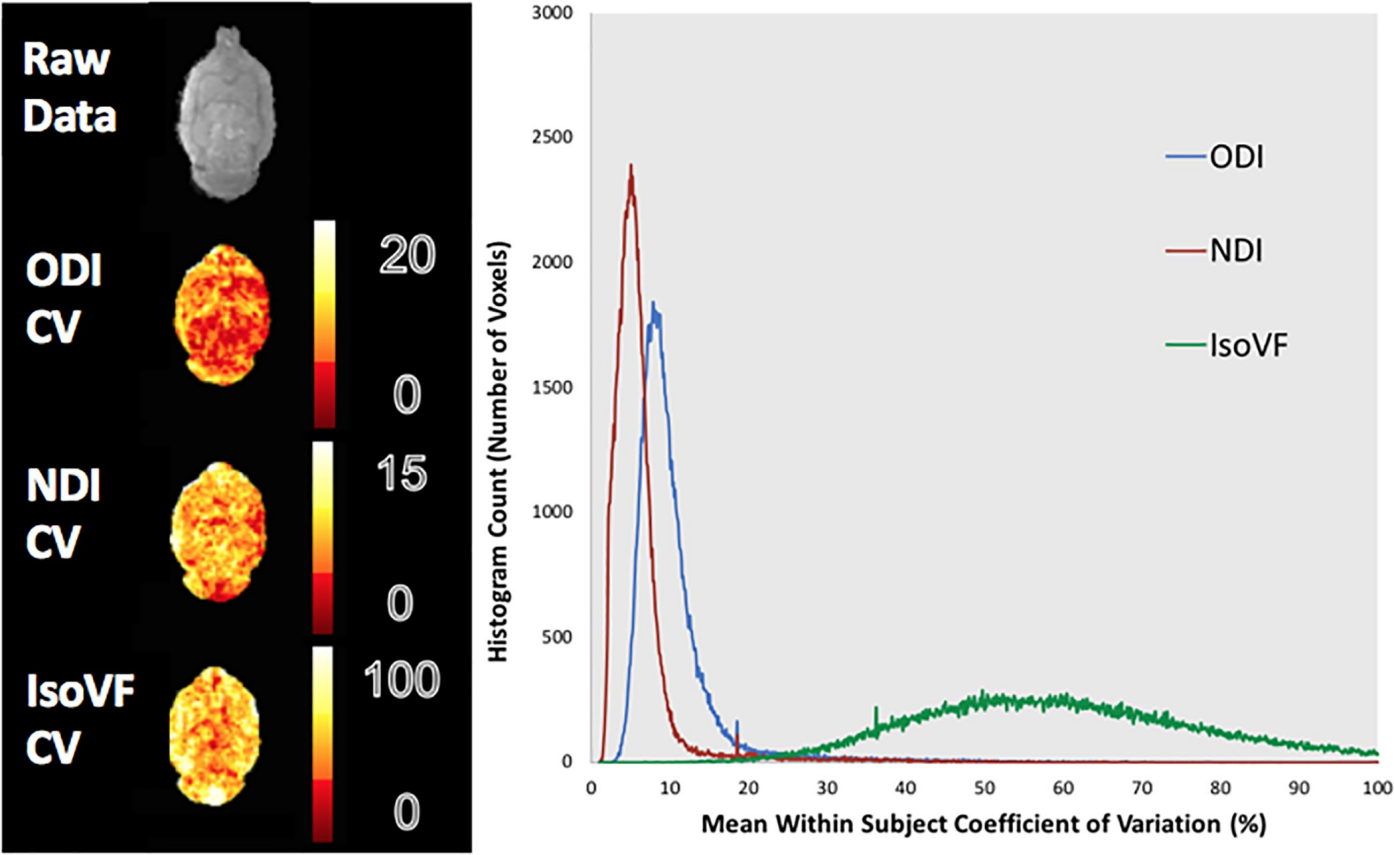
Whole brain average within subject CV maps and histogram. Values for the within subject condition represent the mean CV within each voxel for each subject averaged over all eight subjects. The resulting histogram has been extracted from the averaged scans. Heat maps from a representative slice show the regional variation for each metric.

The whole brain voxel-wise analysis showed a similar trend to the ROI analysis in terms of CVs. In the between subject histogram, over 90% of voxels fell below a CV of 20% for ODI while in the within subject histogram 90% of voxels fell below a CV of 17% (Figs 4 and 5). For NDI over 90% of voxels fell below a CV of 15% and 12% for the between and within subject histograms respectively. The CV for IsoVF, ranged well above 100% for many voxels for both histograms.

### Voxel-wise ROI analysis

**Fig 6:**
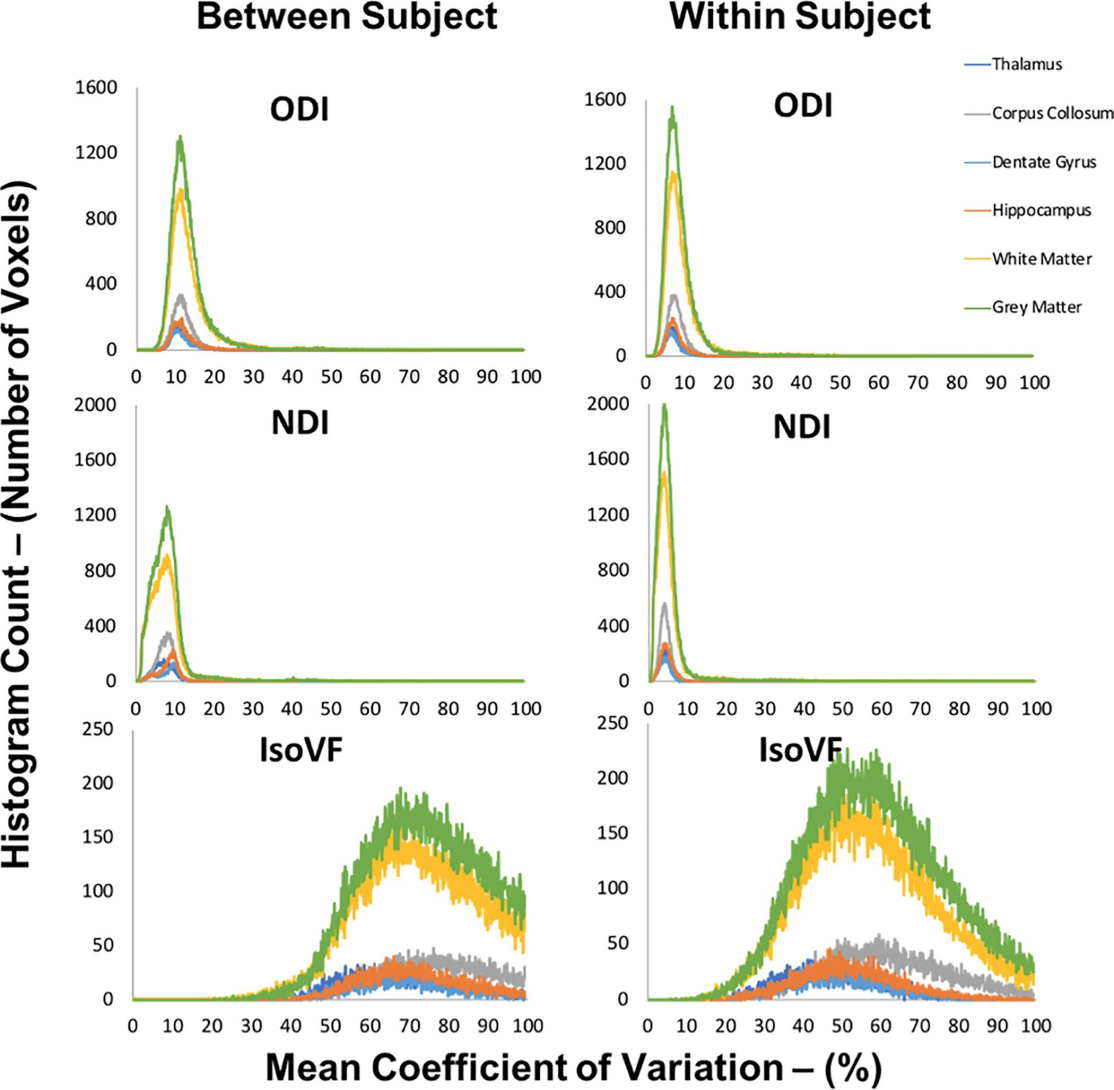
Voxel-wise between and within subject CV histograms within each representative ROI. Voxel-wise values for the between subject condition represents the mean CV within each ROI for both the scan and rescan conditions averaged over the two scans. Voxel-wise values for the within subject condition represent the mean CV within each ROI averaged over the eight subjects.

The voxel-wise approach targeted to specific ROIs (Fig 6) reinforced the results observed in mean ROI and the voxel-wise approaches. For ODI, over 90% of voxels fell below a CV of 18% in the between subject histogram, and 12% in the within subject histogram for all ROIs. For NDI, over 90% of voxels fell below a CV of 10% in the between subject histogram and 8% in the within subject histogram for all ROIs. The CV for IsoVF once again ranged well above 100% for many voxels in all ROIs. In all cases, dispersion of CV values increased with increasing ROI sizes. Likewise, in all cases and all ROIs the CVs and dispersion of CV values were lower within subjects compared to between subjects.

### Sample sizes and minimum detectable effect

**Fig 7:**
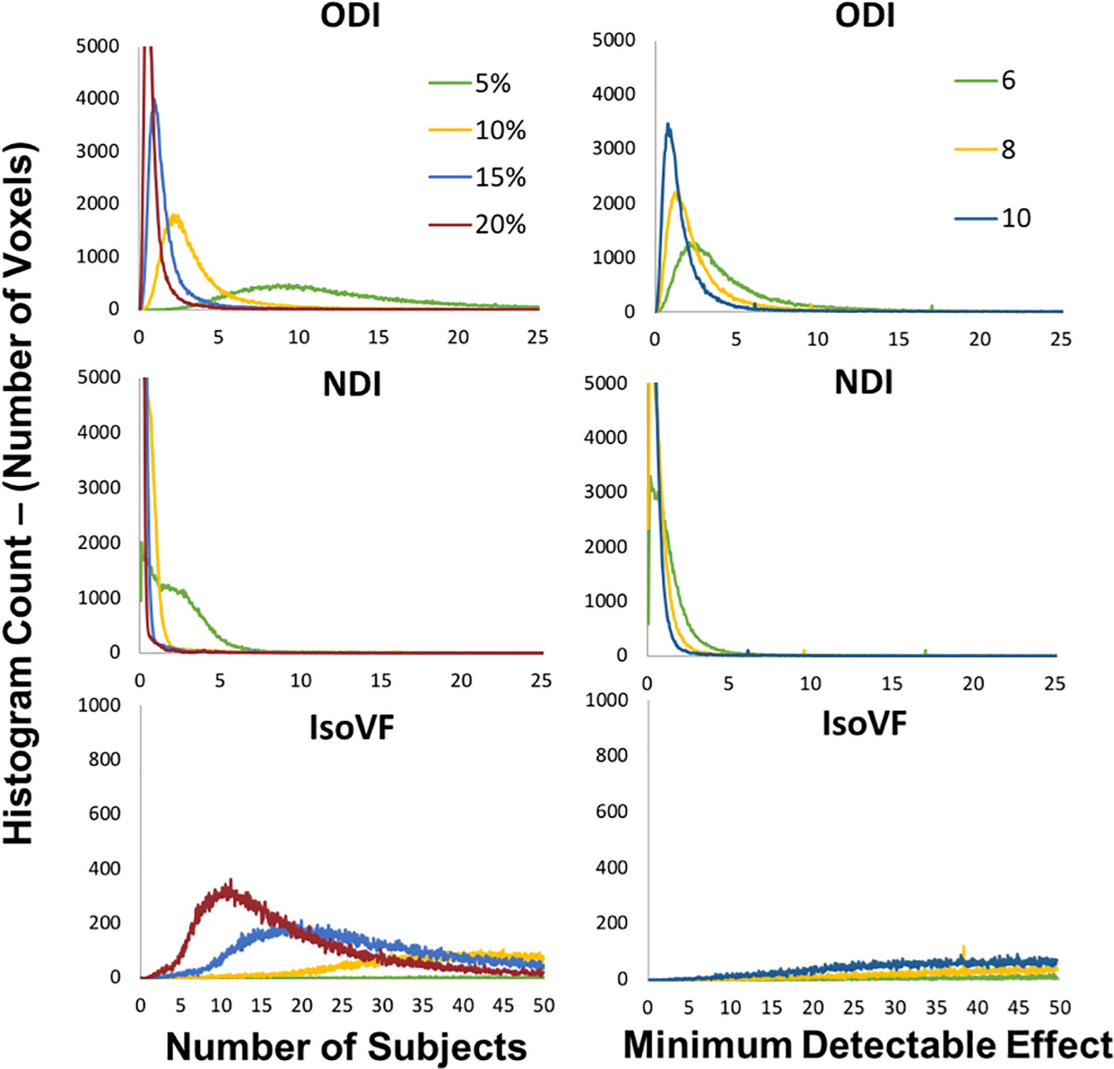
Whole brain voxel-wise histograms representing the i) number of subjects necessary to detect a statistically significant effect with a change in the given metric of 5%, 10%, 15% and 20% and ii) the minimum detectable effect with each metric under a scan-rescan study design given group sample sizes of 6, 8 and 10. Note the varied scales for the IsoVF metric in each category as opposed to the ODI and NDI metrics.

Using the between subject whole brain voxel-wise CVs, the minimum number of subjects was determined on a voxel-by-voxel basis that would allow detection of a statistically significant change of 5%, 10%, 15% and 20% between subjects in each metric. ODI produced detectable changes on the order 10% in all voxels for moderate sample sizes (n < 10) but required large sample sizes (n > 10) for whole brain voxel-wise detection of changes on the order of 5% (Fig 7). NDI was able to detect changes on the order of 5% in all voxels with small sample sizes (n < 6 for all voxels). IsoVF required large sample sizes (n > 10) to detect changes of any magnitude on a voxel-wise basis.

Using the within subject whole brain voxel-wise CVs, the minimum statistically significant change that may be detected in each metric on a voxel-wise basis was determined using a scan re-scan protocol for sample sizes of 6, 8 and 10 within each group. For over 90% of voxels, ODI was also to detect small changes (<10%) on a scan-rescan basis with all sample sizes discussed, NDI showed detection of very small changes (<5%) with all sample sizes discussed and IsoVF lacked the ability to detect significant changes at any samples size explored.

## Discussion

This study examined the reproducibility of the three most commonly derived NODDI metrics (ODI, NDI, and IsoVF) in the rodent brain at 9.4 Tesla. ODI and NDI were reproducible, showing low coefficients of variation in both the between and within subject conditions. CVs were lower within subjects compared to between subjects, indicating less variability on a within subject scan-rescan basis, as expected. These trends were observed in the mean ROI, whole brain voxel-wise, and targeted voxel-wise analyses.

Using the whole brain coefficients of variation on a voxel-by-voxel basis it was possible to detect changes on the order of 10% and 5% respectively in ODI and NDI metrics with feasible study samples sizes. For ODI, over 90% of voxels showed the ability to detect a 10% or greater change with sample sizes of five or more, while NDI showed the ability to detect a change of 5% or greater with sample sizes of five or more. NDI was the most sensitive in all cases, followed by ODI. Furthermore, it was shown that using a scan-rescan protocol and standard sample sizes (6, 8, and 10) it was possible to detect very small changes for both ODI and NDI. For example, sample sizes of eight per group (common to many preclinical studies) allowed biological effects as small as 5% to be detected on a voxel by voxel basis for both metrics.

While ODI and NDI were shown to be reproducible metrics, IsoVF was not. This was shown previously in similar studies of the human brain [25]. The IsoVF metric suffers from not only low values intrinsically in the given context but is also highly susceptible to noise [25]. This combination led to high average CVs (> 20%) in all measures explored in this study. Consequently, with the scan parameters used in this study, the reproducibility of IsoVF is limited. Improvements in SNR could increase the reproducibility of IsoVF but would come at the cost of increased scan time, decreased image resolution, or decreased angular resolution. It should be noted that inaccurate estimates of IsoVF could slightly bias the absolute values of NDI at low SNR, however this bias is expected to be consistent across all subjects and scans, allowing meaningful comparisons to be made under consistent scan parameters. As of now the optimal angular and image resolution in a rodent model of NODDI has yet to be explored, and it must be assumed that higher resolution in both improves the quality of the resulting scalar metric maps. Thus, an increase in scan time would be necessary. As the scan time in the present study was already high (83 minutes) it may be that the necessary scan time to improve the quality of IsoVF scalar maps is not feasible or cost effective.

These findings are consistent with previous research using human subjects. In humans, NODDI has been shown to produce accurate and reproducible metrics of ODI and NDI both between and within subjects [25]. The magnitude of NDI, ODI, and IsoVF have been shown to vary at different field strengths [25], and thus it is important to characterize these metrics not only in new animal models, but also at each field strength used. At the time of writing we are not aware of any other studies that specifically look at the reproducibility of NODDI in an *in-vivo* rodent model. This study shows NODDI to be reproducible in a rodent model at 9.4 Tesla and that this technique has the potential to detect very subtle tissue microstructure changes in a rodent model.

There are several limitations that should be considered in this study. Registration was performed using FLIRT [35] and FNIRT [37] in FSL. The quality of these registrations was not specifically quantified in terms of similarity overlap. As the quality of registration is important to both ROI and voxel-wise analyses, future studies may benefit from improvements and optimization of the registration process. Specifically, when using a targeted voxel-wise approach within a given ROI, registration can be optimized within that region, thereby increasing the precision of the analysis. Currently, the optimal angular resolution, image resolution and b-value combination in our NODDI pulse sequence has not been fully explored in a rodent model at 9.4 Tesla. It is possible that at high angular resolution more subtle changes in orientation are detected, and at higher image resolution more subtle changes in neurite microstructure may be shown. Furthermore, these parameters may vary greatly in coherently ordered white matter compared to less ordered structures within regions of grey matter and may be altered in disease states. These considerations must be balanced against scan time for any *in-vivo* study. Further exploration of optimal angular resolution sampling schemes and image resolution would lend strength to this technique and lead to a more robust acquisition and analysis pipeline. Additionally, the optimal b-value has not been expressly explored in rodents at 9.4 Tesla. In humans, the optimal b-values were explored through simulation and in-vivo study, and it was found that as long as two shells with moderate b-value were used, the precise choice of b-value made minimal difference [15]. Finally, it should be noted that for the within-subject calculation of CV, the standard deviation was determined from only two data points. As a result this this standard deviation may not accurately represent the spread of data within the population, leading to an unknown bias in the resulting CV.

The current study was designed to evaluate reproducibility of the NODDI metrics over a one-week interval, as this interval is relevant for many time course studies. While subtle changes in brain plasticity were of some concern, the results show that over this interval the ODI and NDI metrics were reproducible. Finally, we know of no studies which have expressly attempted to produce a template of absolute values of NODDI metrics within various brain regions of rodents at 9.4 Tesla. While intrinsic variability will always be present in these values due to scan parameters, it would be useful to attempt this characterization for all brain regions.

Preclinical imaging techniques, and specifically diffusion imaging techniques, are designed to detect very subtle changes in disease models, which may not be seen with anatomical based imaging techniques. The potential to improve the ability to detect these very small changes through novel neuroimaging techniques, such as NODDI, could illuminate early events in disease processes such as neurodegeneration. Early detection of key pathways and mechanisms involved in the progression of these devastating diseases may lead to a more thorough understanding of the downstream biological effects. By showing NODDI metrics to be reproducible in a rodent model at ultra-high field strengths, we may now apply this technique to appropriate pre-clinical models, in an effort to further our understanding of complex diseases processes affecting neuroanatomy.

## Supporting information

S1 Dataset Region of interest NODDI metrics and CVs for scan and rescan conditions. (XLSX)

S2 Dataset Voxel-wise within subject CVs and minimum detectable effect data. (XLSX)

S3 Dataset Voxel-wise between subject CVs and minimum number of subjects data. (XLSX)

S1 Supporting Information Optimized registration parameters used for non-linear registration in FSL (FNIRT). (DOCX)
