# Supplementary material for: Reproducibility of Neurite Orientation Dispersion and Density Imaging (NODDI) in rats at 9.4 Tesla
Source: PLoS One. 2019 Apr 29;14(4):e0215974. doi: 10.1371/journal.pone.0215974 (PMC6488046; doi:10.1371/journal.pone.0215974)
Supplement: S1 Supporting Information — (DOCX) [file pone.0215974.s004.docx]

**Reproducibility of Neurite Orientation Dispersion and Density Imaging in Rats (NODDI) at 9.4 Tesla**

**Supplemental Document – FNIRT Registration Parameters**

fnirt --in=input_file --ref=reference_image --imprefm=1 --impinm=1 --imprefval=0 --impinval=0 --subsamp=8,4,2,1 --miter=5,5,10,15 --reffwhm=4,2,0,0 --infwhm=8,4,0,0 --lambda=300,75,30,10 --estint=1,1,1,0 --warpres=5,5,5 --aff=matrix_from_FLIRT –iout=registered_image --cout=registration_coefficients

As stated in the manuscript, the masks were transformed into diffusion space, thus the “registration_coefficients” file noted here was passed to each mask to move it into the diffusion space of each individual subject.

The “registration_image” file was inspected for accuracy. It is noted in the Limitations section that a visual inspection was used to assess the accuracy of the registration.
